# Burden and Risk Factors of Melioidosis in Southeast Asia: A Scoping Review

**DOI:** 10.3390/ijerph192315475

**Published:** 2022-11-22

**Authors:** Kasturi Selvam, Thanasree Ganapathy, Mohamad Ahmad Najib, Muhammad Fazli Khalid, Nor Azlina Abdullah, Azian Harun, Wan Mohd Zahiruddin Wan Mohammad, Ismail Aziah

**Affiliations:** 1 Institute for Research in Molecular Medicine (INFORMM), Health Campus, Universiti Sains Malaysia, Kubang Kerian 16150, Kelantan, Malaysia; 2 School of Health Sciences, Health Campus, Universiti Sains Malaysia, Kubang Kerian 16150, Kelantan, Malaysia; 3 Department of Community Medicine, School of Medical Sciences, Universiti Sains Malaysia, Kubang Kerian 16150, Kelantan, Malaysia; 4 Department of Medical Microbiology and Parasitology, School of Medical Sciences, Health Campus, Universiti Sains Malaysia, Kubang Kerian 16150, Kelantan, Malaysia; 5 Hospital Universiti Sains Malaysia, Jalan Raja Perempuan Zainab 2, Kubang Kerian 16150, Kelantan, Malaysia

**Keywords:** melioidosis, prevalence, risk factors, Southeast Asia

## Abstract

This scoping review aims to provide a comprehensive overview of human melioidosis in Southeast Asia as well as to highlight knowledge gaps in the prevalence and risk factors of this life-threatening disease using available evidence-based data for better diagnosis and treatment. Preferred Reporting Items for Systematic Review and Meta-Analyses Extension for Scoping Reviews (PRISMA-ScR) was used as the guideline for this review. The literature search was conducted on 23 March 2022 through two electronic databases (PubMed and Scopus) using lists of keywords referring to the Medical Subject Headings (MeSH) thesaurus. A total of 38 articles related to human melioidosis were included from 645 screened articles. These studies were carried out between 1986 and 2019 in six Southeast Asian countries: Thailand, Cambodia, Malaysia, Myanmar, Singapore, and Vietnam. Melioidosis has been reported with a high disease prevalence among high-risk populations. Studies in Thailand (48.0%) and Cambodia (74.4%) revealed disease prevalence in patients with septic arthritis and children with suppurative parotitis, respectively. Other studies in Thailand (63.5%) and Malaysia (54.4% and 65.7%) showed a high seroprevalence of melioidosis among Tsunami survivors and military personnel, respectively. Additionally, this review documented soil and water exposure, diabetes mellitus, chronic renal failure, thalassemia, and children under the age of 15 as the main risk factors for melioidosis. Human melioidosis is currently under-reported in Southeast Asia and its true prevalence is unknown.

## 1. Introduction

Melioidosis is a fatal infectious disease that was first recognized in 1911 in Myanmar by the British pathologist Alfred Whitmore and his assistant Krishnaswami [1]. It is caused by the motile, Gram-negative environmental bacterium *Burkholderia pseudomallei*, which is found freely in water and soil, predominantly in moist, slightly acidic, nutrient-rich soil [2]. Primary routes of the acquisition of *B. pseudomallei* infection are considered to be skin inoculation, ingestion, and inhalation [3]. Melioidosis results in a wide spectrum of clinical manifestations, ranging from acute fulminant disease to latent infection with subsequent reactivation [4], and the incubation period of acute melioidosis is 1–21 (mean 9) days [5].

High-risk groups include adults with underlying predisposing medical conditions, among which diabetes is the most prominent, being present in approximately 50.0% of cases [3]. Culture and isolation of *B. pseudomallei* from any clinical specimen remains the mainstay of melioidosis diagnosis. Melioidosis is difficult to diagnose, mainly due to the symptoms’ similarities with those of other diseases, especially infection by causative agents such as *Mycobacterium tuberculosis* (pneumonia) and *Entamoeba histolytica* (liver abscess) [6,7,8,9,10]. Furthermore, reports revealed that one of the major issues is also a lack of understanding in diagnosing and recognizing the possibility of melioidosis among clinicians and laboratory staff [11]. This neglected bacterial infection is frequently under-reported in some countries due to inadequate or non-existent laboratory-based surveillance systems, making it difficult to determine the true prevalence of melioidosis.

The first attempt to estimate the global burden of human melioidosis was made in 2016, with predictions of 165,000 and 89,000 cases and deaths per year, respectively [11]. Melioidosis is endemic across tropical countries, especially in Southeast Asia and Northern Australia [3]. In Northeast Thailand, approximately 2000 culture-confirmed melioidosis cases per year are reported with a case fatality rate (CFR) of 40.0% [12,13]. In Singapore, 550 melioidosis cases have been reported over the last ten years with one-fifth of those cases resulting in death [14]. To date, comprehensive data on the true distribution and burden of melioidosis as well as its potential risk factors are not available in the Southeast Asia region because reports have only been compiled for whole countries.

Determining the overall prevalence of melioidosis in the Southeast Asia region will give a better indication of its distribution and severity. Additionally, understanding the risk factors associated with melioidosis in Southeast Asia can provide complete information for establishing effective preventive strategies. The last narrative review of melioidosis in Southeast Asia was in 2000 [15]. Hence, the present scoping review aims to update our understanding of melioidosis in Southeast Asia by including studies that evaluated the prevalence and risk factors that have occurred since the last review.

## 2. Materials and Methods

The present scoping review utilized the updated Preferred Reporting Items for Systematic Review and Meta-Analyses Extension for Scoping Reviews (PRISMA-ScR) guidelines [16]. PRISMA-ScR aims to guide the reporting of scoping reviews.

### 2.1. Search Strategy

The literature search was conducted on 23 March 2022 through two databases (PubMed and Scopus) using lists of keywords referring to the Medical Subject Headings (MeSH) thesaurus. These keywords were combined using the Boolean operators OR (within key concepts) and AND (between key concepts) as follows: (“melioidosis” OR “*Burkholderia pseudomallei*”) AND (“prevalence” OR “risk factor”) AND (“Malaysia”). A similar search strategy was applied to Brunei, Burma (Myanmar), Cambodia, Timor-Leste, Indonesia, Laos, the Philippines, Singapore, Thailand, and Vietnam. An additional search was conducted by manually screening the references of the retrieved literature.

### 2.2. Selection of Studies

Articles were excluded if (i) the studies did not report the prevalence or risk factors of melioidosis; (ii) the studies were published in languages other than English or Malay; (iii) case series or reports, qualitative studies, conference papers, proceedings, abstract-only articles, editorial reviews, letters of communications, commentaries, systematic reviews, studies conducted outside of Southeast Asia, studies on non-living subjects such as soil and water properties, and study designs with non-random sampling. The retrieved literature was downloaded into the Endnote reference manager, and duplicates were identified and removed from this review. The references were distributed to four authors (K.S., T.G., M.F.K., and N.A.A.), who independently reviewed all the articles for the title and abstract screening. A satisfactory agreement for the screening process was assessed between the authors. Discrepancies between the authors were solved through a discussion with another author (M.A.N).

### 2.3. Data Extraction

Two authors (K.S. and T.G.) independently performed full-text screening and summarized the findings. The data included the year of study, study area, study design, sample size, prevalence (disease prevalence and seroprevalence), risk factor and diagnostic assay, and target population. Another three authors (A.H., W.M.Z.W.M., and I.A.) verified the screening results and checked the scoping review manuscript.

## 3. Results

### 3.1. Search Results

**Figure 1:**
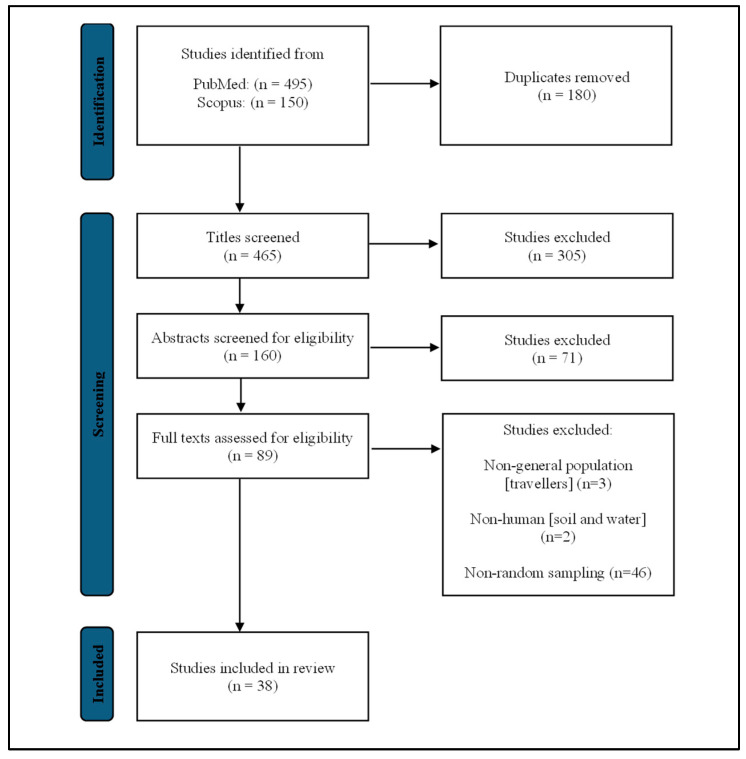
PRISMA-ScR flow diagram showing the process of selecting studies.

Screening from two databases resulted in a total of 645 studies, and 180 duplicates were removed from the collection. After screening the titles and abstracts of the remaining 465 articles, 376 studies were then excluded because the articles were not relevant to this review. A total of 51 studies were excluded from 89 selected articles during full-text screening, leaving 38 studies in the review that fulfilled the inclusion and exclusion criteria of this study (Figure 1). The characteristics of the final 38 studies were summarized in Table 1, Table 2 and Table 3.

### 3.2. Prevalence of Melioidosis

The prevalence of melioidosis in Southeast Asia was classified into disease prevalence and seroprevalence as shown in Table 1 and Table 2, respectively.

#### 3.2.1. Disease Prevalence of Melioidosis

**Figure 2:**
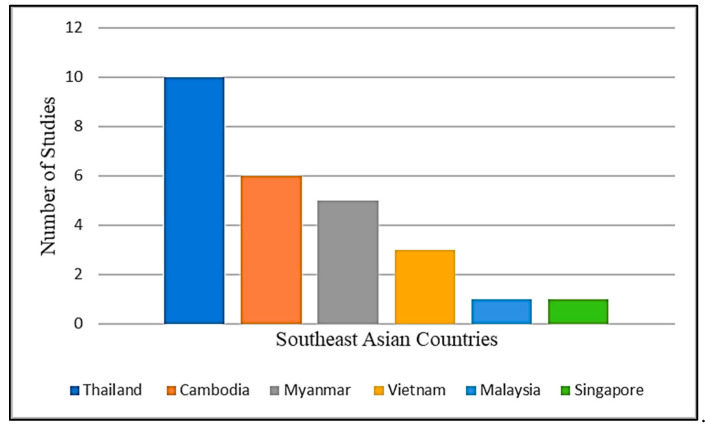
The number of studies on the prevalence of melioidosis conducted in Southeast Asia countries.

Disease prevalence of melioidosis has been determined in six Southeast Asia countries, as shown in Figure 2: (i) Thailand (10); (ii) Cambodia (6); (iii) Myanmar (5); (iv) Vietnam (3); (v) Malaysia (1); and (vi) Singapore (1). In Thailand, the prevalence of melioidosis has been studied primarily in the northeast region. A high prevalence of melioidosis was reported in Thailand (48.0%) and Cambodia (74.4%) among high-risk populations, such as patients with septic arthritis and children with suppurative parotitis, respectively [22,29]. In Cambodia, studies on the prevalence of melioidosis were carried out mostly in the provinces of Takeo, Kampong Cham, and Siem Reap. Melioidosis prevalence in Myanmar was reported in the range of 0.33 to 5.7% between 2004 and 2019, with most of these studies taking place in Yangon. Overall, the disease prevalence of melioidosis in Southeast Asia ranged from 0.02 to 74.4%, as determined by diagnostic tests such as culture, antigen detection, and molecular assays using blood, sputum, synovial fluid, and pus samples.

#### 3.2.2. Seroprevalence of Melioidosis

**Figure 3:**
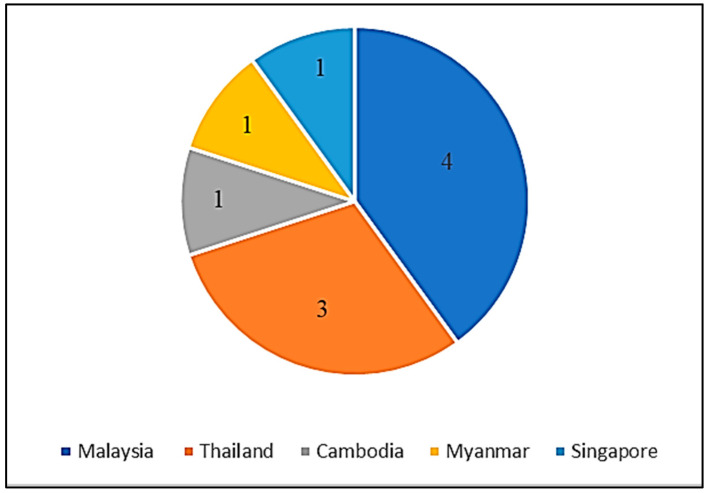
The number of studies on the seroprevalence of melioidosis conducted in Southeast Asia countries.

Seroprevalence of melioidosis has been determined in five Southeast Asia countries, as shown in Figure 3: (i) Malaysia (4); (ii) Thailand (3); (iii) Cambodia (1); (iv) Myanmar (1); and (v) Singapore (1). In Thailand, a high prevalence of melioidosis (63.5%) was reported among Tsunami survivors using an indirect haemagglutination assay (IHA) with a cut-off value of ≥1:10 [44]. Melioidosis seroprevalence in Myanmar reported in 2016 showed that it often occurred in the delta region during the rainy season. In Malaysia, military personnel were found to have a high melioidosis prevalence compared to other populations [48,49,50]. A high seroprevalence of melioidosis (71.4%) was reported in Singapore among patients with radiological evidence of splenic abscess [51]. Overall, the seroprevalence of melioidosis in Southeast Asia ranged from 3.2 to 71.4%, as determined by antibody detection assays such as the enzyme-linked immunosorbent assay (ELISA), IHA, and immunofluorescent assay (IFA).

### 3.3. Risk Factors of Melioidosis

**Figure 4:**
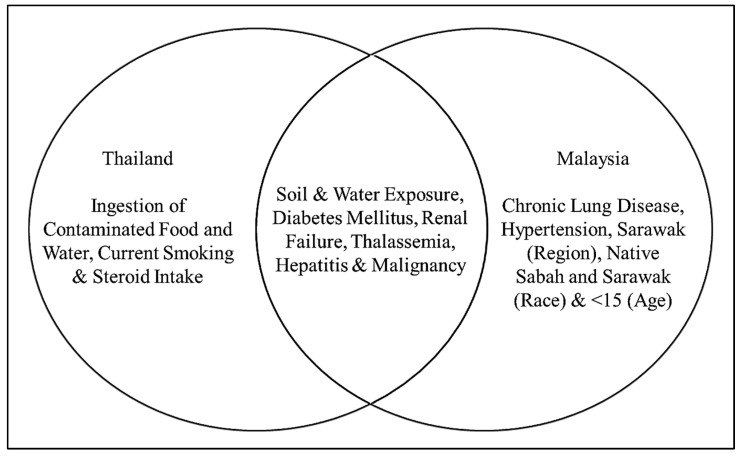
Risk factors of melioidosis were identified among the population in Thailand and Malaysia.

A total of five studies evaluated the risk factors of melioidosis. All studies used logistic regression analysis (simple and multiple) to determine the significant risk factors. Variables with an odds ratio (OR) of more than 1.0 indicate a higher risk for melioidosis. In Thailand, two studies were conducted with a total sample size of 1380 individuals, utilizing the culture method, while in Malaysia, three studies were performed with a total sample size of 44,383 individuals, employing culture as well as antibody detection assays. In Thailand, the major risk factors are soil and water exposure (OR = 3.5), diabetes mellitus (OR = 5.9), and thalassaemia disease (OR = 10.2). In the Malaysia, Sabah (OR = 1.61), and Sarawak (OR = 1.75) regions, pre-existing diseases such as diabetes mellitus (OR = 3.46) and chronic renal failure (OR = 4.04) and an age of less than 15 years (OR = 4.71) are primary risk factors. A study conducted in Thailand reported a higher prevalence of melioidosis in patients with grouped risk factors (i. diabetes and occupation involving high soil and water exposure (OR = 8.5); ii. diabetes and occupation involving moderate soil and water exposure (OR = 5.6)) compared to those with a single risk factor (i. high soil and water exposure (OR = 3.3); ii. moderate soil and water exposure (OR = 2.1); iii. diabetes mellitus (OR = 5.9)). As indicated in Figure 4, some risk factors are common in both countries, such as exposure to water and soil, diabetes, chronic renal failure, thalassemia, hepatitis, and cancer.

## 4. Discussion

*B. pseudomallei* is a tier 1 select agent that causes melioidosis, a disease that has been threatening human life for more than a century. It is often found in soil and surface water in Southeast Asia and northern Australia; nevertheless, melioidosis case reports and predictive modelling studies imply that it is likely widespread across the tropics [3]. The global mortality burden of melioidosis each year is greater than that of other well-known diseases, such as leptospirosis and dengue fever [11,55]. However, melioidosis has not been listed as a neglected tropical disease as defined by the World Health Organization (WHO).

In most countries, poor reporting systems have masked the true burden of this deadly disease, which is predicted to be substantially greater than what is reported [56]. Putting this into context, Thailand has developed initiatives to strengthen the surveillance and reporting system, as seen with the establishment of the Thailand Melioidosis Network in 2012. This network highlighted that melioidosis is still prevalent in some parts of Thailand. The success of the reporting system may be seen in the number of fatal melioidosis cases voluntarily reported each year in Thailand, which increased from 10 cases per year in 2014 to 112 in 2015 and 100 in 2016 [13].

Prevalence is the percentage of people in a population who have a specific disease or characteristic at a given time or over a given period [57]. The current review highlighted the disease and seroprevalence of melioidosis in Southeast Asia countries. Melioidosis is highly endemic to Northeast Thailand, which explains why most studies were carried out in this region [13]. The high disease prevalence of melioidosis (48.0%) observed among patients with septic arthritis [22] indicated that septic arthritis is one of the clinical manifestations of melioidosis in the endemic area; thus, clinicians in this area should be aware of this and request microbiological tests. Interestingly, a high seroprevalence (76.9%) of melioidosis was reported in tsunami survivors in 2004 in the Phangnga area of Southern Thailand due to contamination of wounds and/or inhalation of *B. pseudomallei* contained in aerosolized mud or water particles post-tsunami [44]. However, this study highlighted that the high prevalence was due to the low cut-off value of the IHA test (≥1:10) compared to the cut-off value of ≥1:160.

Additionally, high disease prevalence (74.4%) was found among children with potential suppurative parotitis in Cambodia [29]. The parotid form of melioidosis is typically associated with the ingestion of *B. pseudomallei* in the water supply and its colonization of the parotid duct. In Myanmar, the majority of studies were conducted in Yangon, the city where melioidosis was first identified. A recent review also stated that most of the melioidosis cases were reported in Yangon [58]. In Vietnam, three studies revealed the prevalence of melioidosis. According to a recent analysis, melioidosis cases increased in Vietnam after a bilateral project called Research Network on Melioidosis and *Burkholderia pseudomallei* (RENOMAB) was established [59]. This project has shown that melioidosis is widely distributed throughout the country, with North-Central Vietnam potentially having a high endemicity rate [60]. Several agricultural-based states in Malaysia were highlighted in this review, such as Kedah, Pahang, Sabah, and Sarawak. A recent study stated that melioidosis infection had the highest prevalence in Sarawak. The majority of people living in Sarawak are involved in environmental-related occupations, such as farming, forestry, and fishing, leading to more *B. pseudomallei* exposure and subsequent infection [49]. Military personnel in Sabah and Sarawak had the highest seroprevalence (Table 2). The majority of them were from the infantry division, which is known for conducting intensive military drills in rice fields, filthy waterways, and muddy terrain [50]. The previous serosurvey conducted in Malaysia from 1964 to 1966 revealed a 7.3% seropositivity with the highest rates in recruits from Kedah and Sabah [61].

Despite rapid urbanization, melioidosis still exists in Singapore with an overall annual incidence of 0.6–2.4 per 100,000 between the years 2000 and 2015 [62]. A study reported a seroprevalence of 71.4% among patients with radiological evidence of splenic abscess. This finding relates to a previous study that found deep organ abscesses are one of the most common systemic manifestations of melioidosis in Singapore, accounting for 40.7% of cases, and that the frequency of deep organ abscesses is on the rise [62]. Another analysis revealed that, even though Singapore is a highly urbanized environment with little soil contact, the frequency of melioidosis cases is linked to higher rainfall totals and humidity levels in the weeks leading up to illness development. The finding suggests that water, rather than soil, may be the primary vehicle for *B. pseudomallei* infection transmission and acquisition [14].

In addition to the prevalence of melioidosis in Southeast Asia, the current review provides information on potential risk factors. In Thailand and Malaysia, occupational exposure to contaminated soil and water is one of the leading causes of melioidosis. *B. pseudomallei* is most abundant in the soil at depths of ≥10 cm from the surface; however, during the rainy season, it can move from deeper soil layers to the surface, where it can then multiply [3,63]. It was perceived that farmers were more exposed to *B. pseudomallei*-contaminated soil and flood water while working in the paddy fields during the monsoon season [64]. This is made worse by the fact that *B. pseudomallei* may immediately penetrate open wounds or abrasions on farmers. Apart from Thailand and Malaysia, recent environmental surveys indicate that over 80.0% of soil samples in Southern Vietnam are positive for *B. pseudomallei* [59]. Therefore, residents, rice farmers, and visitors should use protective equipment (such as boots and gloves) and cover open skin wounds, cuts, or burns if they must come into close contact with soil or water. Furthermore, outdoor exposure to dust clouds has been linked to the development of melioidosis in Thailand. This is because continuous rain causes water levels to rise, causing *B. pseudomallei* to build on the soil surface and serve as a reservoir for aerosolized bacteria inhalation [65]. *B. pseudomallei* is rarely found in the air, although its DNA was discovered in filtered air using quantitative PCR [66]. Hence, high-risk individuals should avoid going outside after heavy rain, particularly in rural regions, as a self-preventive strategy of melioidosis.

Additionally, diabetes mellitus was found to be the most common risk factor for melioidosis in case–control studies conducted in Thailand and Malaysia. This finding was supported by a recent study in Perak, Malaysia, which found that 71.1% of patients have diabetes mellitus [67]. Patients with diabetes mellitus were three times more likely to be infected by melioidosis than non-diabetic patients [68]. Interestingly, diabetes mellitus with soil and water exposure results in higher OR values compared with single variables, which highlights those individuals exposed to more risk factors are highly vulnerable to *B. pseudomallei* infection. Diabetic patients are assumed to be less capable of killing or inactivating *B. pseudomallei*, which may be driven by defective neutrophil phagocytosis, decreased migration in response to interleukin 8, and an inability to delay apoptosis/necrosis [69]. Melioidosis can be exacerbated by renal disease and thalassemia. These two medical diseases are linked to a malfunctioning innate immune system, specifically the function of macrophages and neutrophils and increased chances of *B. pseudomallei* infection [26,70]. Malaysian paediatric cohorts reveal that up to 40.0% of cases are thalassemia major. Chelation therapy, on the other hand, appears to have reduced infection rates [71].

Moreover, drinking contaminated water is also considered a contributor to the occurrence of melioidosis. *B. pseudomallei* is also commonly found in unchlorinated water supplies and drinking water in rural areas in Thailand [72]. Therefore, water should be chlorinated and boiled before being consumed, and drinking water directly from shallow wells, lakes, rivers, ponds, and streams should be avoided. Other underlying risk factors include consuming contaminated food, steroid intake, gender, and ethnicity. Prediction of melioidosis risk factors is critical since it not only allows the public to take preventative measures to prevent infection but also aids medical practitioners and healthcare workers in diagnosing and prescribing early treatment for the disease.

The present study has several limitations, such as (i) small sample size, (ii) the variety of diagnostic assays used to detect *B. pseudomallei* in patients’ samples, (iii) heterogeneity of study population, (iv) no critical appraisal of individual studies, (v) lack of recent studies, and (vi) no studies on the prevalence and risk factors in the other five Southeast Asia countries (Brunei, Timor-Leste, Indonesia, Laos, and the Philippines). It is wise to note that the number of subjects in a study has a direct impact on the reliability and precision of the results. Certain studies in this review had a small sample size. For instance, a study conducted in Singapore included 21 patients with radiological evidence of splenic abscess, with a prevalence of 71.4% [51]. This could induce bias and lead to an overestimation of the magnitude of the association. As a result, it is preferable to conduct a larger confirmatory study to estimate realistic prevalence and risk factors, because large studies produce smaller confidence intervals and more precise estimates [73].

Another key limiting factor is the diagnostic assays employed to detect *B. pseudomallei*. The selected studies mainly utilized culture and antibody detection methods compared to antigen detection and molecular assays. Culture, antigen detection, and molecular techniques were employed in order to detect melioidosis clinically, while antibody detection assays were developed to confirm exposure to *B. pseudomallei*. The utilization of different diagnostics techniques makes it difficult to determine the true prevalence of melioidosis from the included studies. The gold standard for melioidosis diagnosis is culture, which is highly specific but has a low sensitivity (60.0%); thus, there is a considerable risk of false-negative results [74]. Repeating cultures (particularly of blood, sputum, urine, and pus samples) should be performed in patients with strong indications of *B. pseudomallei* infection. It is common for subsequent samples to be positive despite initial negative results [75]. The culture-based studies may indicate incidence which has been labelled as prevalence is a potential weakness of the review. Moreover, antigen detection assays such as the AMD test provide faster and highly specific results with the disadvantage of poor sensitivity, especially in blood samples. This is due to the lower number of the *B. pseudomallei* or its components in blood samples [76]. Moreover, molecular methods such as PCR and matrix-assisted laser desorption/ionization time-of-flight mass spectrometry (MALDI-TOF MS) enable rapid results with high sensitivity and specificity compared to the diagnosis of *B. pseudomallei* using the culture method [77]. MALDI-TOF MS is an accurate and discriminatory tool for the identification of *B. pseudomallei* [78].

Furthermore, a variety of assays for detecting antibodies against *B. pseudomallei* have been developed, but most of them are based on poorly defined antigens and have never been internationally standardized or subjected to significant critical evaluation. In some endemic areas, background seropositivity rates in the healthy population are extremely high, owing to recurrent exposures to *B. pseudomallei* or closely related organisms [79]. Thus, serology has limited use in endemic areas as many people will be positive based on exposure and unfortunately, most of the studies used serologic methods to define the prevalence of melioidosis. Standardization of the method, including the cut-off value, is also necessary to avoid overestimation of outcomes because each diagnostic methodology has a different accuracy in terms of sensitivity and specificity. The included studies used different cut-off values to determine the prevalence. For example, the employed cut-off values of IFA-IgM were 1:8, ≥1:20, ≥1:80, and ≥1:160.

Importantly, the sensitivity of diagnostic assays is greatly influenced by melioidosis status. Melioidosis infection can be acute, chronic, and latent, and only individuals who develop clinical symptoms (either acute or chronic) are considered to have melioidosis [3]. Because of its low sensitivity, IHA results for acute infections are more likely to be negative; however, IHA may help support a diagnosis of chronic infection [80]. On the other hand, in acute infections, bacteria can be cultured easily compared to chronic infections. Another drawback of this review is that it was difficult to generalize the prevalence of melioidosis due to the heterogeneity of the study population. In some studies, the prevalence of melioidosis is high because high-risk individuals were recruited as the study subjects, such as patients with septic arthritis, children with suppurative parotitis, patients with radiological evidence of splenic abscess, tsunami survivors, and military personnel. Another limitation is that the review did not include a critical appraisal of individual studies. Furthermore, the lack of recent studies is another limitation of this review. For example, the selected studies were conducted in Singapore and Vietnam between 2005 and 2013 [39,51]. There are no studies of prevalence and risk factors for the other five Southeast Asian countries. As a result, estimating the prevalence and risk factors of the disease in individual countries, as well as Southeast Asia as a whole, is difficult. Despite its limitations, this is the first scoping review that systematically summarizes previously published data on the prevalence and risk factors of melioidosis in Southeast Asia. Melioidosis is not only more common in these countries, but it is also expected to become more prevalent as a result of the global pandemic of diabetes, which increases the risk of melioidosis by at least 12 times and is primarily increasing in low- and middle-income tropical countries [81,82].

## 5. Conclusions

In conclusion, this review emphasizes that the epidemiology of melioidosis in Southeast Asia is unknown. The prevalence of melioidosis is most likely underestimated due to the poor surveillance and reporting systems as well as the inadequacy of local and extensive surveys conducted in endemic regions. Furthermore, melioidosis is considered a non-notifiable disease in a few Southeast Asian countries, including Malaysia, Indonesia, and Laos. Therefore, it is critical to strengthen the surveillance programmes in Southeast Asian countries as well as throughout the world. This will lead to the identification of risks related to *B. pseudomallei* infection and the development of effective disease prevention, control, diagnosis, and treatment strategies.

## Figures and Tables

**Table 1:** Summary of the included studies for disease prevalence of melioidosis.

No.	Year of Study	Study Area	Study Design	Sample Size (n)	Prevalence (%)	Diagnostic Assay	Target Population	Reference
1.	1986–1987	Thailand (Northeast)	CS	225	24.7	Blood culture	Patients with CAS	[17]
2.	2007–2008	Thailand (Central)	CS	221	2.7	Blood culture	Patients infected with NFGNB	[18]
3.	2002–2014	Thailand (South)	CS	145	11.0	Culture	Patients with suspected melioidosis	[19]
4.	2009–2013	Thailand (Northeast)	CS	2031	28.3	Blood culture	Patients had a presumed pathogen isolated	[20]
5.	2012–2013	Thailand (Northeast)	CS	118	2.5	Sputum culture	Patients with suspected pulmonary tuberculosis	[21]
6.	2012–2014	Thailand (Northeast)	CS	154	48.0	Blood and synovial fluid culture	Culture-confirmed bacterial septic arthritis patients	[22]
7.	2013–2015	Thailand	CS	463	0.5	Multiplex real-time PCR (whole blood)	Patients with a fever (agricultural communities)	[23]
8.	2013–2017	Thailand (Northeast)	CS	4989	3.0	Blood culture	Patients with community-acquired infection	[24]
9.	2014–2019	Thailand	CS	41	2.4	Pus culture	Patients with SCL	[25]
10.	NR	Thailand (Northeast)	CS	211	1.4	Blood and pus culture	Patients with NTDT	[26]
11.	2006–2009	Cambodia	CS	9821	0.02	Blood culture	Patients with AFI	[27]
12.	2007–2010	Cambodia (Takeo and Kampong Cham)	CS	2407	1.6	Blood and sputum culture	Patients with ALRI	[28]
13.	2007–2011	Cambodia (Siem Reap)	CS	39	74.4	Parotid culture	Children with suppurative parotitis	[29]
14.	2009–2010	Cambodia (Siem Reap)	CS	1180	0.5	Blood culture	Febrile children	[30]
15.	2014–2015	Cambodia (Takeo)	CS	139	5.0	Blood and sputum culture	Patients with SIRS	[31]
16.	2015	Cambodia (Kampong Cham)	CS	404	1.0	Sputum culture	Patients with suspected tuberculosis	[32]
17.	2004–2006	Myanmar (Yangon)	CS	133	2.3	Pus culture	Patients with abscess	[33]
18.	2015–2016	Myanmar (Yangon)	CS	90	1.1	Blood culture, VITEK2, and LA	Patients with BSI	[34]
19.	2016–2017	Myanmar (Yangon)	CS	120	0.83	Blood culture	Patients with fever and dysfunction of at least two organ systems	[35]
20.	2017–2019	Myanmar (Yangon)	CS	364	5.7	Culture, LA, API 20 NE, antibiotic susceptibility testing and molecular confirmation	Patients infected with oxidase-positive Gram-negative rods	[36]
21.	2018–2019	Myanmar (Yangon)	CS	299	0.33	Sputum culture and antigen detection (AMD-LFA)	Patients presenting with fever and productive cough	[37]
22.	2016	Malaysia (Pahang)	CS	336	6.0	Blood, sputum, tissue, pus aspirate, and body fluid aspirate culture	Patients with AFI	[38]
23.	1992–1998	Vietnam (South)	CS	3653	0.25	Blood culture	Hospitalized patients	[39]
24.	2005–2009	Vietnam (North)	CS	7428	0.7	Blood culture	Patients with bacteraemia	[40]
25.	2011–2013	Vietnam (North)	CS	738	2.0	Blood culture	Patients with BSI	[41]
26.	2003–2005	Singapore	CS	80	5.0	Respiratory specimen culture	Patients admitted to the medical ICU with CAP	[42]

n: number of samples; CS: cross-sectional; NR: not reported; PCR: polymerase chain reaction; LA: latex agglutination; AMD-LFA: active melioidosis detect–lateral flow assay; CAS: community-acquired septicaemia; NFGNB: non-fermentative Gram-negative bacteria; SCL: suppurative cervical lymphadenitis; NTDT: non-transfusion dependent thalassemia; ALRI: acute lower respiratory infections; AFI: acute febrile illness; BSI: bloodstream infections; ICU: intensive care unit; SIRS: systemic inflammatory response syndrome; CAP: community -acquired pneumonia; API20NE: Analytical Profile Index 20E.

**Table 2:** Summary of the included studies for seroprevalence of melioidosis.

No.	Year of Study	Study Area	Study Design	Sample Size (n)	Prevalence (%)	Diagnostic Assay	Target Population	Reference
1.	1992	Thailand (Northeast)	CS	439	5.0	Antibody detection (IHA 1:160; IFA-IgM 1:8 and IFA-IgG 1:32)	Culture-positive tuberculous patients	[43]
2.	2005	Thailand (South)	CS	52	63.5 (≥1:10) and 13.5 (≥1:160)	Antibody detection (IHA ≥1:10 and ≥ 1:160)	Tsunami survivors	[44]
3.	2002–2014	Thailand (South)	CS	145	11.0	Antibody detection (IFA-IgM ≥ 1:20 and IHA ≥ 1:160)	Patients with suspected melioidosis	[19]
4.	2005 and 2014–2015	Cambodia	CS	1316	12.0	Antibody detection (ELISA-IgG using OPS 1: 2000)	Patients with fever or sepsis of unknown origin	[45]
5.	2016	Myanmar (delta region during the rainy season)	CS	124	3.2	Antibody detection (ELISA using OPS and Hcp1 1: 2000)	Febrile patients	[46]
6.	2010	Malaysia (Pahang)	CS	153	7.2	Antibody detection (IFA-IgM ≥ 1:80 or four-fold rise in IgM)	People involved in the search and rescue operation for a drowned victim with a fever	[47]
7.	2013–2014	Malaysia	CS	17,234	10.5	Antibody detection (IFA-IgM using the whole-cell antigen ≥ 1:160)	Patients with suspected melioidosis	[48]
8.	2015–2019	Malaysia	CS	26,665	16.4	Antibody detection (IFA-IgM ≥ 1:160 and ELISA-IgM ≥ 1:320)	Patients with suspected melioidosis	[49]
9.	NR	Malaysia (Sabah and Sarawak)	CS	420	54.4 (exotoxin) and 65.7 (whole-cell antigens)	Antibody detection (ELISA using exotoxin and whole-cell antigens)	Military personnel	[50]
10.	1996–2005	Singapore	CS	21	71.4	Antibody detection	Patients with radiological evidence of splenic abscess	[51]

n: number of samples; CS: cross-sectional; NR: not reported; IHA: indirect haemagglutination assay; IFA: immunofluorescent assay; OPS: O-polysaccharide; ELISA: enzyme-linked immunosorbent assay; Hcp1: haemolysin co-regulated protein.

**Table 3:** Summary of the included studies for risk factors of melioidosis.

No.	Year of Study	Study Area	Study Design	Sample Size (n)	Risk Factor (OR)	Diagnostic Assay	Target Population	Reference
1	1997	Thailand (Northeast)	CC	580	High soil and water exposure (3.3) Moderate soil and water exposure (2.1) Diabetes mellitus (5.9) Hematologic or solid tumour (0.4) Pre-existing renal disease (2.9) Thalassaemic disease (10.2) Diabetes and occupation involving high (8.5) and moderate soil and water exposure (5.6)	Blood, urine, sputum, pus, and throat swab culture	Patients with suspected bacteraemia	[52]
2	2010–2011	Thailand (Northeast)	CC	800	Working in a rice field (2.1) Other activities involving exposure to soil or water (1.4) Open wound (2.0) Eating food contaminated with soil or dust (1.5) Drinking untreated water (1.7) Outdoor exposure to dust clouds (1.3) Outdoor exposure to rain (2.1) Water inhalation (2.4) Current smoking (1.5) Steroid intake (3.1)	Blood culture	Hospitalized patients	[53]
3	2005–2011	Malaysia (Kedah)	CC	484	Diabetes mellitus (3.46) Chronic renal failure (4.04) Other diseases (chronic lung failure, HIV, and immunocompromised states) (1.2)	Blood and other body fluids culture	Hospitalized patients	[54]
4	2013–2014	Malaysia	CS	17,234	Gender: female (1.12)	Antibody (IgM) detection (IFA ≥ 1:160)	Patients with suspected melioidosis	[48]
					Age group: <15 (4.71); 15–24 (2.82); 25–34 (2.21); 35–4; (2.05); and 45–54 (1.62)			
					Ethnicity: Malay (0.92); Indians (1.20); Orang Asli (1.17); Sabah native (1.61); Sarawak native (1.27); and non-Malaysian (1.37)			
					Region: northern (1.32); southern (1.28); eastern coast (1.47); Sabah (0.90); and Sarawak (1.46)			
5	2015–2019	Malaysia	CS	26,665	Gender: female (1.05)	Antibody (IgM) detection (IFA ≥ 1:160 and ELISA ≥ 1:320)	Patients with suspected melioidosis	[49]
					Age group: <15 (3.04); 15–24 (2.99); 25–34 (2.59); 35–44 (1.98); and 45–54 (1.32)			
					Region: northern (1.17); southern (0.91); eastern (1.17); Sabah (1.61); and Sarawak (1.75)			
					Race: Malay (1.27); Indian (1.23); Orang Asli (1.78); Bumiputera Sarawak (2.04); Bumiputera Sabah (1.90); and others (1.52)			
					Previous exposure: history of swimming in the river (4.39) and history of soil contact (1.02)			
					Underlying diseases: diabetes mellitus (0.73); hypertension (0.60); chronic kidney disease (0.56); thalassemia (1.34); chronic lung disease (3.60); hepatitis (4.43); retroviral disease (1.31); and malignancy (1.27)			

n: number of samples; CC: case–control; OR: odds ratio; HIV: human immunodeficiency virus; IFA: immunofluorescent assay; ELISA: enzyme-linked immunosorbent assay.

## Data Availability

Not applicable.
